# Risk Factors and Birth Outcomes of Anaemia in Early Pregnancy in a Nulliparous Cohort

**DOI:** 10.1371/journal.pone.0122729

**Published:** 2015-04-15

**Authors:** Gwinyai Masukume, Ali S. Khashan, Louise C. Kenny, Philip N. Baker, Gill Nelson, SCOPE Consortium

**Affiliations:** 1 School of Public Health, Faculty of Health Sciences, University of the Witwatersrand, Johannesburg, South Africa; 2 Irish Centre for Fetal and Neonatal Translational Research (INFANT), Department of Obstetrics and Gynaecology, University College Cork, Cork, Ireland; 3 Department of Epidemiology and Public Health, University College Cork, Cork, Ireland; 4 Gravida: National Centre for Growth & Development, Liggins Institute, University of Auckland, Auckland, New Zealand; Shanghai 1st Maternity and Infant hospital of Tongji University, CHINA

## Abstract

**Background:**

Anaemia in pregnancy is a major public health and economic problem worldwide, that contributes to both maternal and fetal morbidity and mortality.

**Objective:**

The aim of the study was to calculate the prevalence of anaemia in early pregnancy in a cohort of ‘low risk’ women participating in a large international multicentre prospective study (n = 5 609), to identify the modifiable risk factors for anaemia in pregnancy in this cohort, and to compare the birth outcomes between pregnancies with and without anaemia in early gestation.

**Methods:**

The study is an analysis of data that were collected prospectively during the Screening for Pregnancy Endpoints study. Anaemia was defined according to the World Health Organization’s definition of anaemia in pregnancy (haemoglobin < 11g/dL). Binary logistic regression with adjustment for potential confounders (country, maternal age, having a marital partner, ethnic origin, years of schooling, and having paid work) was the main method of analysis.

**Results:**

The hallmark findings were the low prevalence of anaemia (2.2%), that having no marital partner was an independent risk factor for having anaemia (OR 1.34, 95% CI 1.01-1.78), and that there was no statistically significant effect of anaemia on adverse pregnancy outcomes (small for gestational age, pre-tem birth, mode of delivery, low birth weight, APGAR score < 7 at one and five minutes). Adverse pregnancy outcomes were however more common in those with anaemia than in those without.

**Conclusion:**

In this low risk healthy pregnant population we found a low anaemia rate. The absence of a marital partner was a non-modifiable factor, albeit one which may reflect a variety of confounding factors, that should be considered for addition to anaemia’s conceptual framework of determinants. Although not statistically significant, clinically, a trend towards a higher risk of adverse pregnancy outcomes was observed in women that were anaemic in early pregnancy.

## Introduction

Anaemia is a state where the delivery of oxygen to the tissues is impaired because of a quantitative or qualitative deficiency of haemoglobin or red blood cells [1]. According to the World Health Organization (WHO), anaemia in pregnancy occurs when, at sea level, the haemoglobin is < 11 g/dL or the haematocrit is < 33%, regardless of gestation [2,3].

The United States Centers for Disease Control and Prevention’s (CDC) definition of anaemia in pregnancy differs from the WHO definition only in the second trimester where the cut off haemoglobin is < 10.5 g/dL and haematocrit is < 32% [4].

Despite these definitions, anaemia in pregnancy is not quite straight forward, because of the physiologic changes that occur during pregnancy, which also involve the haematologic system [5]. Some authorities maintain that additional variables of altitude, cigarette smoking and ethnicity may alter the definition of anaemia in individuals [6,7]. Others are of the opinion that altitude should not modify the definition [8] and that there is insufficient information to alter the definition of anaemia based on ethnicity [3].

These subtleties of defining anaemia in pregnancy are important as they have implications for comparisons between studies and between populations. Stephens et al. reported that approximately 38% of pregnant women worldwide are anaemic [9]. The estimated prevalence of anaemia in pregnancy differs widely between continents, being highest in Africa (55.8%) and Asia (41.6%), and lowest in Europe (18.7%) and North America (6.1%) [10]. In general, as pregnancy progresses, the prevalence of anaemia increases [11].

Anaemia in pregnancy is a major public health and economic problem worldwide and contributes to both maternal and fetal morbidity and mortality; anaemia of pregnancy can also have profound short-term and far-reaching sequelae for the newborn [12–14]. Anaemia, even in early pregnancy has been associated with adverse pregnancy outcome [15]. Clinical manifestations include fetal growth restriction, preterm delivery, low birth weight [16], impaired lactation, poor maternal/infant behavioural interactions, post partum depression and increased fetal and neonatal mortality [12,13]. Economic losses occur because iron deficiency anaemia has been associated with decreased work capability of adults and reduced cognitive function of children that may persist into adulthood; impaired motor development a manifestation of anaemia also adds to economic loss [9,17].

**Fig 1:**
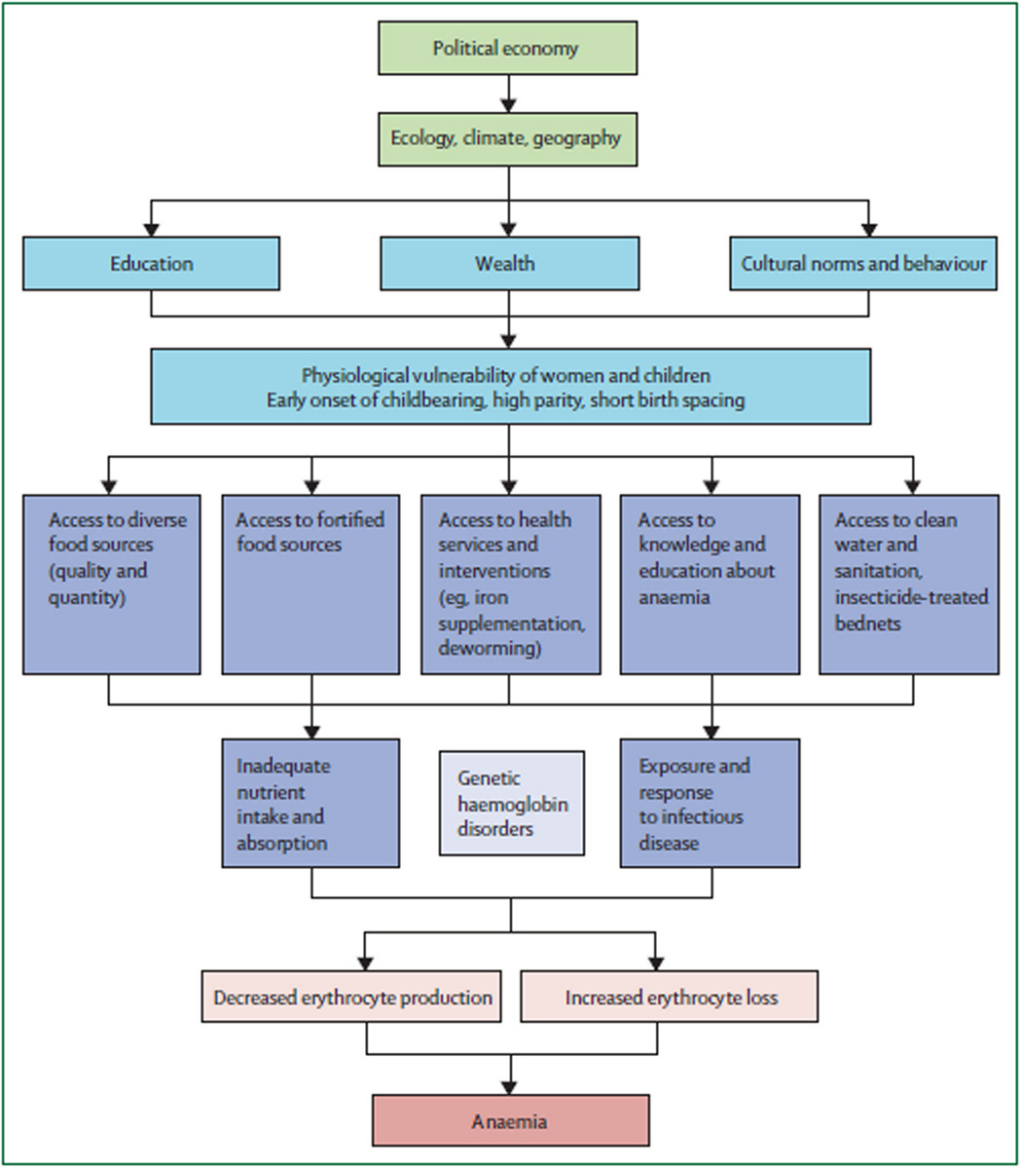
Conceptual model of the determinants of anaemia, reproduced with permission from Balarajan et al. [18].

The risk factors/determinants/causes of anaemia can be found at multiple interacting levels [18].The immediate causes of anaemia can be considered to be decreased red blood cell/haemoglobin production and increased loss of red blood cells/haemoglobin, as a result of nutritional, infectious and genetic influences. Some of the important risk factors include deficiency of nutrients such as iron (apparently the most common risk factor), folate and vitamin B_12_, infections such as human immunodeficiency virus (HIV), malaria and hook worms, and disorders in the structure or production of haemoglobin such as sickle cell disease and the thalassemias [12,13]. The aetiology of anaemia in pregnancy can be considered in the broader context of Balarajan and colleagues’ “Conceptual model of the determinants of anaemia” (see Fig 1), whereby alterations in the political economy, climate, ecology, cultural norms, etc. eventually culminate in decreased red blood cell/haemoglobin production and increased loss. Other risk factors in the conceptual model include teenage pregnancy, ‘low’ educational level, ‘poor’ socioeconomic status, a short inter pregnancy interval and high parity [18–20]. In aggregate, nutritional, infectious and genetic risk factors for anaemia are less common in high income than in low and middle income countries [21].

Impaired delivery of oxygen to tissues appears to be the central mechanism by which anaemia increases the risk of maternal organ (brain, heart, kidney) injury and mortality [22]. Oxygen delivery to the uterus (and fetus) may be reduced in pregnant women with anaemia because of this impaired tissue oxygen delivery [23].

The primary aims of this study were to determine the modifiable risk factors for anaemia in early pregnancy, and to compare the pregnancy outcomes between women with and without anaemia in early pregnancy.

## Materials and Methods

The SCOPE (SCreening fOr Pregnancy Endpoints) study is an international, prospective, multicentre cohort study of 5 690 ‘low-risk’ nulliparous women with singleton pregnancies in four high income countries: New Zealand, Australia, England and Ireland (www.scopestudy.net/). The primary aim of the SCOPE study is to develop screening tests to predict pre-eclampsia, fetal growth restriction and spontaneous preterm births in a low risk population.

Details of the SCOPE study methods have been published in detail elsewhere [24] but in brief, recruitment of participants into the study started in Auckland, New Zealand in November 2004 and finished in Cork, Ireland in February 2011. The participants ranged in age from 16 to 45 years and were recruited into the study before 15 weeks’ gestation, through community midwives, general practitioners, hospital antenatal clinics, obstetricians and self referral. Women’s partners, along with their newborn infants, are also involved in the study. Women were excluded if they: 1) were considered to be at high risk of pre-eclampsia, fetal growth restriction or spontaneous preterm birth due to underlying medical conditions (chronic hypertension, diabetes, renal disease, systemic lupus erythematosus, anti-phospholipid syndrome, sickle cell disease, HIV), previous cervical knife cone biopsy, ≥ 3 previous terminations or ≥ 3 miscarriages, current ruptured membranes; 2) had a major uterine anomaly, a known major fetal anomaly or abnormal karyotype; or 3) received an intervention that could modify pregnancy outcome (e.g. aspirin therapy, cervical suture).

At 14–16 weeks’ and 19–21 weeks’ gestation, participants were interviewed and examined by a research midwife, and underwent blood and urine tests. An ultrasound scan was performed at 19–21 weeks. Participants were followed prospectively, with pregnancy outcome data and baby measurements collected by research midwives.

Ethical approval was obtained from local ethics committees [New Zealand AKX/02/00/364, Australia REC 1712/5/2008, London and Manchester 06/MRE01/98 and Cork ECM5(10)05/02/08] and all women provided written informed consent. For the secondary analysis of the SCOPE study dataset, presented in this paper, ethics approval was sought from the Human Research Ethics Committee (Medical) of the University of the Witwatersrand M130966.

Prior to analysis, each participant’s measured booking haemoglobin (obtained typically in the first trimester) was adjusted to take into account cigarette smoking during three months pre-pregnancy up to and including the first study visit at 14–16 weeks (data on smoking was available for this time period). The adjustment was made according to the WHO criteria [3,6] (see Table 1). Because smoking has effects which can persist via epigenetic changes [25], and haematological indices can take years to return to normal after smoking cessation [26], an adjustment to the measured haemoglobin was made considering the entire period for which data on smoking was available. The adjusted haemoglobin was then used to classify participants as having anaemia or not according to the WHO definition of anaemia in pregnancy. No adjustment to the haemoglobin for altitude was made because all the participating SCOPE centres were below 1 000m above sea level.

**Table 1:** Criteria for the adjustment of haemoglobin due to smoking cigarettes.

Smoking status*	Measured haemoglobin adjustment (g/dL)
Non-smoker	0
< 10 cigarettes	0
≥10-<20 cigarettes	-0.3
≥ 20-<40	-0.5
≥40 cigarettes	-0.7

* Number of cigarettes smoked per day: < 10 cigarettes, < ½ packet; ≥10-<20 cigarettes, ½-1 packet; ≥ 20-<40 cigarettes, 1–2 packets; ≥40 cigarettes, ≥2 packets

We investigated the following risk factors for their association with anaemia: maternal age, country, marital status, ethnicity, schooling, paid work, body mass index, maternal socioeconomic index, fruit consumption, vegetable consumption, folate intake, iron and mineral intake, alcohol, psychological scales, paternal age and socioeconomic index. We investigated the following outcomes for their association with anaemia: small for gestational age, preterm delivery, mode of delivery, low birth weight and APGAR score. The variables were examined as presented in Table 2.

**Table 2:** Comparison of participants without and with anaemia.

Characteristic	Not anaemic	Anaemic^a^	p-value^b^
	n = 5 484	n = 125	
	n (%)	n (%)	
**Maternal**			
Age (years), median IQR^c^	29 (25–32)	28 (22–33)	0.2359
Teenager	391 (7.1)	14 (11.2)	0.082
Country			0.008
Australia	1 130 (20.6)	30 (24.0)	
Ireland	1 742 (31.8)	28 (22.4)	
New Zealand	1 980 (36.1)	42 (33.6)	
United Kingdom	632 (11.5)	25 (20.0)	
Haemoglobin (g/dL), median IQR^c^	12.8 (12.3–13.4)	10.7 (10.4–10.8)	< 0.001*
No marital partner	518 (9.5)	20 (16.0)	0.014
Ethnic origin			< 0.001*
European	4 947(90.2)	97 (77.6)	
Asian	160 (2.9)	9 (7.2)	
Indian	125 (2.3)	9 (7.2)	
Polynesian	113 (2.1)	2 (1.6)	
Other (including African)	139 (2.5)	8 (6.4)	
Schooling ≤ 12 years	2 054 (37.4)	63 (50.4)	0.003
No paid work at 15 weeks visit	795 (14.5)	29 (23.2)	0.007
Body mass index (kg/m2)(ethnicity adjusted,evaluated at 14–16 weeks)			0.583
< 18.5	82 (1.5)	2 (1.6)	
≥ 18.5– < 25	2 998 (54.7)	76 (60.8)	
≥ 25.0 –< 30	1 564 (28.5)	31 (24.8)	
≥ 30	840 (15.3)	16 (12.8)	
Socioeconomic index, median IQR^c^	45 (28–50)	43 (27–50)	0.3058
High fruit intake a month before conception (consumption ≥ 3 times per day)	1 234 (22.5)	23 (18.4)	0.277
High green leafy vegetable intake a month before conception (consumption ≥ 3 times per day)	329 (6.0)	7 (5.6)	0.852
Folate (supplementation in pill form)			
no intake before pregnancy	2 267 (41.3)	72 (57.6)	< 0.001*
no intake during first trimester	2 19 (4.0)	7 (5.6)	0.366
Iron or mineral (supplementation in pill form)			
no intake before conception	3 502 (96.5)	70 (92.1)	0.043
data missing	1 854 (33.8)	49 (39.2)	
no intake during first trimester	3 454 (95.0)	64 (84.2)	< 0.001*
data missing	1 849 (33.7)	49 (39.2)	
Alcohol (units), > 14 units per week (1 unit = 8g pure alcohol)			
3 months pre-pregnancy	602 (11.0)	15 (12.0)	0.718
First trimester	342 (6.2)	6 (4.8)	0.510
Psychological scales(evaluated at 14–16 weeks)			
Edinburgh Postnatal Depression Score ≥10	1 435 (26.3)	41 (33.6)	0.069
data missing	21 (0.4)	3 (2.4)	
Short form State-Trait Anxiety Inventory Score > 90th centile	433 (7.9)	14 (11.5)	0.154
data missing	27 (0.5)	3 (2.4)	
Perceived Stress Scale Score > 90th centile	483 (8.9)	18 (14.8)	0.024
data missing	30 (0.5)	3 (2.4)	
**Paternal**			
Age (years), median IQR^c^	31 (27–35)	30 (25–34)	0.0575
data missing	1 215 (22.2)	33 (26.4)	
Socioeconomic index, median IQR^c^	44 (29–50)	44 (29–50.5)	0.8451
data missing	1 215 (22.2)	33 (26.4)	
**Pregnancy outcome**			
Small for gestational age (<10th percentile for customized birthweight centiles)	617 (11.3)	13 (10.5)	0.778
data missing	20 (0.4)	1 (0.8)	
Preterm delivery (< 37 completed weeks)			
All	347 (6.3)	9 (7.2)	0.698
data missing	14 (0.3)	0 (0)	
Spontaneous	227 (4.2)	7 (5.6)	0.423
data missing	14 (0.3)	0 (0)	
Mode of delivery			0.307
Unassisted vaginal	2 477 (45.3)	47 (37.9)	
Operative vaginal	1 444 (26.4)	35 (28.2)	
Pre-labour Caesarean section	486 (8.9)	11 (8.9)	
Caesarean section in labour	1 058 (19.4)	31 (25.0)	
data missing	19 (0.3)	1 (0)	
Low birth weight (< 2500g)	282 (5.2)	9 (7.3)	0.300
data missing	27 (0.5)	1 (0.8)	
APGAR score at 1 minute < 7	496 (9.2)	16 (12.9)	0.159
data missing	87 (1.6)	1(0.8)	
APGAR score at 5 minutes < 7	60 (1.1)	1 (0.8)	0.754
data missing	88 (1.6)	2 (1.6)	

^a^ The presence or absence of anaemia was adjusted for smoking

^b^ Pearson’s χ^2^ test or Fisher's exact test

^c^ Mann-Whitney test

* p-value < 0.002 was considered statistically significant

### Statistical analysis

All statistical analysis was conducted using Stata version 13IC (StataCorp LP College Station, TX). Continuous variables were tested for normality using histograms and inverse normal plots. The continuous variables were described using the mean (standard deviation—SD) if normally distributed or median (interquartile range—IQR) if not normally distributed.

Frequency (n) and percent (%) were used to report categorical variables. To compare categorical variables, Pearson’s Chi-squared or Fisher’s exact test was used, where appropriate. For the comparison of normally distributed continuous variables, Student’s t-test (two-sample t-test) was used; for non-normally distributed data, the Mann-Whitney test was used.

Kaplan-Meier (KM) curves were plotted, depicting anaemic and non-anaemic participants with regard to their time to delivery. The logrank test was used to ascertain the equality of survivor functions with p-value < 0.05 being considered statistically significant. Although the median gestational ages could have been compared in each anaemia group, KM (survival) plots give a visual depiction and they are less of a summary than the medians.

Two-tailed p-values were reported. Because of multiple testing (30 tests were planned), in order to reduce the chances of a false positive result (type 1 error), Bonferonni’s method (0.05 ÷ 30) was used, giving an adjusted significance threshold of p <0.002.

It has been suggested that methods using forward stepwise selection (or backward selection or a combination of both forward and backward selection) based on pre-determined p-value criteria are not optimal [27]. A better approach to determine which variables to include or exclude in the multivariable logistic regression model is by using external clinical judgment, which is the approach that was adopted in this analysis [27]. The models were adjusted for country, maternal age, having a marital partner, ethnic origin, years of schooling, and having paid work, the *a priori* variables. Participant data may not have been independent of the SCOPE centres. To take account of this, the cluster option in Stata was used.

A sensitivity analysis was conducted where all the participants with a missing booking haemoglobin were assumed to be anaemic.

## Results

**Fig 2:**
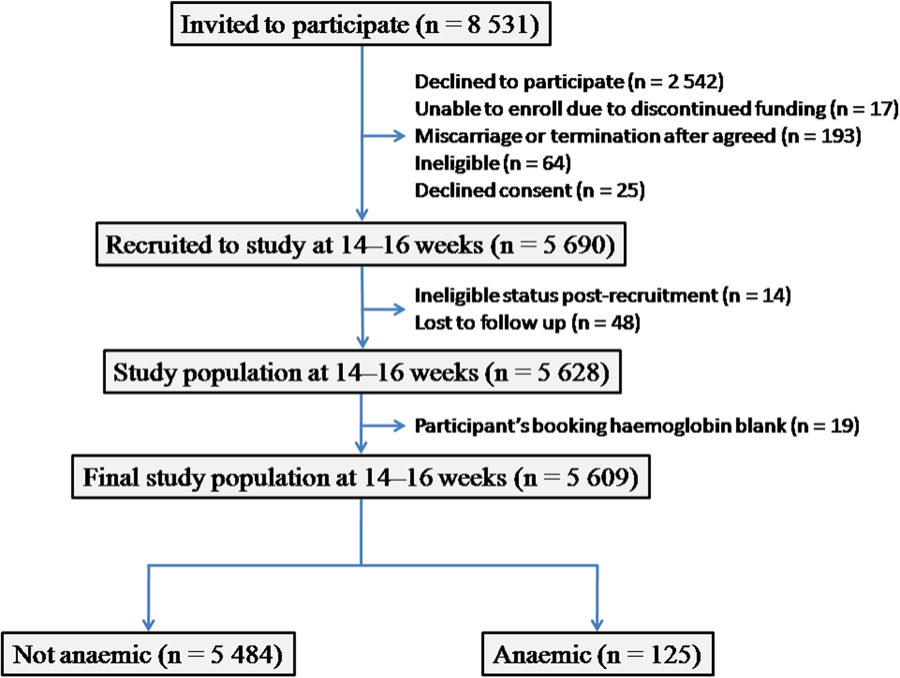
Participant flow chart, adapted from McCarthy et al. [28].

Fig 2 depicts the inclusion of participants in the final analysis; 5 690 participants were recruited into the SCOPE study at 14–16 weeks (STROBE Statement: S1 Table). Forty-eight participants (0.8%) were lost to follow-up and 14 (0.2%) were ineligible after recruitment. Nineteen (0.3%) of the 5 628 remaining participants did not have a booking haemoglobin and were excluded, resulting in a final study population of 5 609 participants at 14–16 weeks. Without adjustment to the measured haemoglobin, 103 (1.8%) of the participants were anaemic; 125 (2.2%) were found to be anaemic after adjusting for cigarette smoking.

Table 2 shows the differences between participants with and without anaemia. The median haemoglobin for those without anaemia was 12.8 g/dL (IQR 12.3–13.4); for those with anaemia it was 10.7 g/dL (IQR 10.4–10.8). Factors that were significantly associated with having anaemia were ethnic origin, reporting folate intake before pregnancy and no iron or mineral intake in the first trimester.

In the final model, which was of good fit, the variables that were independently associated with anaemia in early pregnancy were country, ethnic origin and having a marital partner (Table 3). Assuming that the 19 participants without a booking haemoglobin were anaemic lead to having paid work and reporting folate intake prior to pregnancy being protective of anaemia in early pregnancy.

**Table 3:** Factors associated with anaemia in early pregnancy.

Characteristic	Unadjusted OR (95% CI)	p-value	Adjusted OR (95% CI)	p-value
Country				
Australia	Reference		Reference	
Ireland	0.61 (0.61–0.61)	< 0.001	0.83-(0.68–1.01)	0.060
New Zealand	0.80 (0.80–0.80)	< 0.001	0. 93 (0.78–1.10)	0.410
United Kingdom	1.49 (1.08–2.06)	0.016	1.34 (1.06–1.69)	0.016*
Has marital partner	Reference		Reference	
No marital partner	1.83 (1.28–2.61)	0.001	1.34 (1.01–1.78)	0.044*
Ethnic origin				
European	Reference		Reference	
Asian	2.87 (1.63–5.031)	< 0.001	2.25 (1.19–4.25)	0.012*
Indian	3.67 (2.54–5.32)	< 0.001	3.36 (2.42–4.67)	< 0.001*
Polynesian	0.90 (0.61–1.35)	0.615	0.71 (0.61–0.84)	< 0.001*
Other (including African)	2.94 (1.72–5.02)	< 0.001	1.75 (1.30–2.35)	< 0.001*
Schooling > 12 years	Reference		Reference	
Schooling ≤ 12 years	1.70 (1.39–2.08)	< 0.001	1.26 (0.92–1.72)	0.156
No paid work	Reference		Reference	
Paid work	0.56 (0.43–0.73)	< 0.001	0.73 (0.49–1.07)	0.108
No folate intake before pregnancy	Reference		Reference	
Folate intake before pregnancy	0.52 (0.34–0.80)	0.003	0.64 (0.40–1.03)	0.066
Edinburgh postnatal depression score				
< 10	Reference		Reference	
≥ 10	1.42 (1.09–1.85)	0.009	1.06 (0.82–1.36)	0.654
Perceived stress scale				
≤ 90th centile	Reference		Reference	
> 90th centile	1.78 (0.99–3.21)	0.054	1.47 (0.73–2.96)	0.282
Maternal age (years)	0.97 (0.94–1.00)	0.038	1.02 (0.99–1.05)	0.180

N = 5 575 for adjusted model (The model was adjusted for country, maternal age, having a marital partner, ethnic origin, years of schooling, and having paid work)

OR (Odds Ratio), CI (Confidence Interval)

* p-values < 0.05 were considered statistically significant

Birth outcomes were similar for anaemic and non-anaemic women (Table 4). The pregnancy outcome findings suggested a trend towards a higher risk of adverse pregnancy outcomes for some, but not all outcomes.

**Table 4:** Pregnancy outcomes and anaemia status.

Characteristic	Unadjusted OR (95% CI)	p-value	Adjusted OR (95% CI)*	p-value
Small for gestational age				
Anaemic	0.92 (0.53–1.61)	0.752	0.85 (0.50–1.46)	0.560
Pre-term birth (All)				
Anaemic	1.10 (0.61–2.00)	0.752	1.05 (0.57–1.94)	0.864
Pre-term birth (Spontaneous)				
Anaemic	1.37 (0.53–3.57)	0.519	1.30 (0.48–3.50)	0.610
Mode of delivery (unassisted vaginal—base outcome)^#^				
Operative vaginal				
Anaemic	1.28 (0.69–2.36)	0.434	1.47 (0.75–2.87)	0.259
Pre-labour Caesarean section				
Anaemic	1.19 (0.68–2.09)	0.536	1.40 (0.74–2.62)	0.299
Caesarean section in labour				
Anaemic	1.54 (0.81–2.95)	0.189	1.73 (0.86–3.46)	0.123
Low birth weight				
Anaemic	1.44 (0.67–3.07)	0.351	1.31 (0.62–2.76)	0.485
APGAR score at 1 minute < 7				
Anaemic	0.68 (0.39–1.19)	0.178	0.71 (0.41–1.24)	0.232
APGAR score at 5 minutes < 7				
Anaemic	1.37 (0.19–10.02)	0.755	1.47 (0.21–10.3)	0.699

(The models were adjusted for country, maternal age, having a marital partner, ethnic origin, years of schooling, and having paid work)

OR (Odds Ratio), CI (Confidence Interval)

Reference group—not anaemic

^#^ Relative risk ratio, multinomial logistic regression

* p-values < 0.05 were considered statistically significant

**Fig 3:**
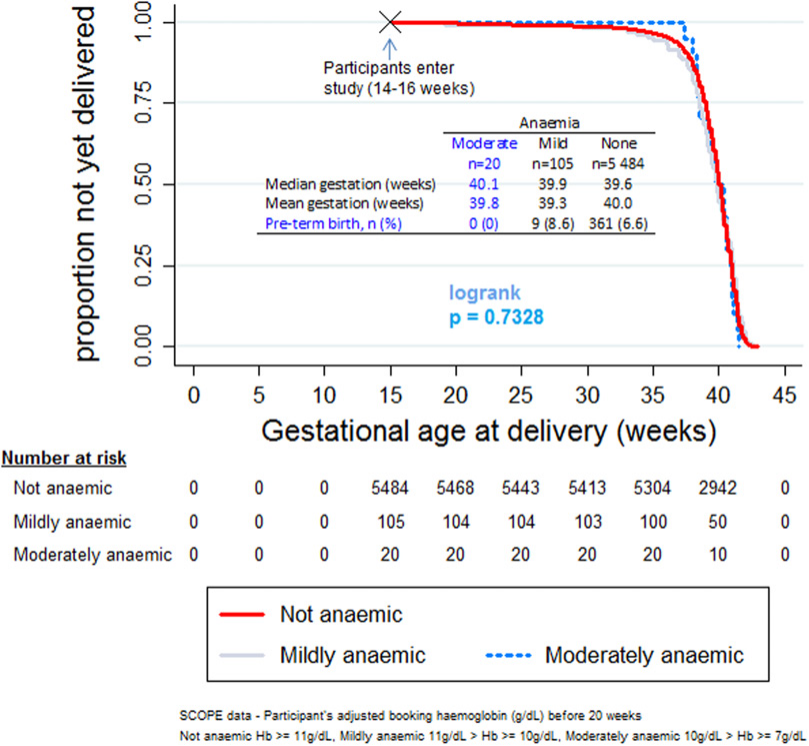
Kaplan-Meier plot estimates of anaemia status and gestational age at delivery. (Note the ‘curious’ behaviour of women with moderate anaemia who have no pre-term birth and are on average—median and mean—delivering later than women with mild anaemia and even those with no anaemia—median.).

Although 76.0% of participants did not have data on serum ferritin, 12 (0.88%) participants with data on serum ferritin were found to have iron deficiency anaemia (defined as serum ferritin < 12μg/L and haemoglobin < 11g/dL [15]). The median gestational age at delivery was similar for the anaemic and non-anaemic women: 38.9 and 40.0 weeks, respectively (see Fig 3), logrank test: p > 0.05. Participants with moderate anaemia had a mean and median gestational age at delivery of 39.8 and 40.1 weeks respectively while the corresponding values for mildly anaemic participants were 39.3 and 39.9 weeks. There was no correlation between the adjusted haemoglobin and birth weight, correlation coefficient 0.0017, p-value = 0.8972.

## Discussion

In this contemporary, large multicenter cohort of nulliparous women in their first ongoing pregnancy we found a very low prevalence of anaemia. The 2.2% prevalence of anaemia in the SCOPE cohort differs sharply with the 22% prevalence reported from high-income regions in recent literature [9]. One possible explanation for this is that SCOPE participants were selected to be ‘low risk’ and were all nulliparous. It is well recognised that co-morbidities such as high parity and short birth interval can affect anaemic status [18]. Furthermore, despite the multicenter nature of the cohort, the ethnicity of SCOPE participants was homogeneous with 89.9% of European ancestry. We did not adjust our findings for ethnic specific variations in haemoglobin concentration because of the low numbers of non-Caucasian participants and because there is sparse data on how to adjust the haemoglobin for ethnicity [7], this may also partially explain our findings. Mandatory folic acid supplementation does not explain the lower incidence of anaemia in this cohort because at the time of patient recruitment none of the participating countries had mandatory folic acid supplementation programs [29].

In the SCOPE cohort, not having a marital partner was associated with higher odds of having anaemia in early pregnancy. This is not surprising because there is evidence that involvement of fathers during pregnancy is associated with diminished negative maternal behaviours and better neonatal outcomes [30]. In addition, not having a partner suggests that the pregnancy was unintended and therefore women did not take steps to optimize their health prior to pregnancy. In general, marriage protects pregnancy [31].

In the adjusted analysis, United Kingdom participants had a higher odds ratio of anaemia in early pregnancy compared to the other countries. It is difficult to untangle the disparate potential contributions of political economy, ecology, geography and climate, all of which are found within the conceptual framework of anaemia’s determinants.

Previous studies mainly from low and middle income countries have shown an association between low education [18,20], the Edinburgh postnatal depression score (the depression being linked to folic acid deficiency [32,33]) and teenage pregnancy [19], but we did not find this in our study.

In our study, from a statistical significance viewpoint, anaemia was not associated with adverse pregnancy outcomes. However, adverse pregnancy outcomes tended to be more common in those with anaemia than in those without. Low birth weight and preterm delivery were similar between pregnant women with and without anaemia in early pregnancy. This is at odds with findings from a recent comprehensive systematic review and meta-analysis [15]. The low prevalence of anaemia in this study (with small numbers of relevant pregnancy outcomes for anaemic participants), due to the deliberate recruitment of ‘low risk’ women, could possibly explain the absence of an effect of anaemia on these adverse pregnancy outcomes.

Contrary to the finding that anaemia prevalence is consistently higher in those of lower socioeconomic status and in those with low body weight [34], in this study the prevalence of anaemia was similar across paternal and maternal socioeconomic groupings and body weight.

Although confirmation of iron deficiency in pregnancy is difficult [13], iron deficiency anaemia is reportedly the most common cause of anaemia in pregnancy. Relatively easy access to iron in fortified cereals and other food products (important sources of iron in industrialized countries) [35], irrespective of socioeconomic status, may partly explain this lack of association.

Although there were few participants with moderate anaemia, they paradoxically delivered later, on average, than participants with mild anaemia (of lesser severity). In fact, none of the participants with moderate anaemia had pre-term labour. This finding contrasts with the u-shaped relationship described in literature (where pre-term birth is more common at both very high and very low maternal haemoglobin concentrations and is uncommon at normal haemogloin levels) [8]. Our finding could be spurious given the small number of participants; however the finding is biologically plausible because paradoxical results have been found in transfusion studies where individuals with more severe anaemia do better [36,37]. We are by no means suggesting that women be made to have moderate anaemia as moderate anaemia seems to be associated with a longer pregnancy compared to mild anaemia, but the finding is worth noting because it can generate hypothesis about underlying biological mechanisms and reveal potential therapeutic targets.

The strengths of this study include its large multi-country prospective cohort design with excellent follow-up where outcome data were available for approximately 99% of the participants. Inclusion of parental infant trios and the availability of a large number of clinical variables further strengthened the study. Stringent real time data monitoring helped to ensure the quality of the data.

The primary study was designed to develop predictive biomarkers for three late pregnancy conditions, and not specifically to answer the question posed in the study reported in this paper.

‘Healthy’ nulliparous women with singleton pregnancies recruited into the SCOPE study are not representative of the general pregnant population. The primary study was conducted in high income countries, thus risk factors for anaemia in pregnancy such as malaria and hook worm infection (which are more common in low and middle income countries) are unlikely to be identified as significant causes of anaemia. In addition, women with HIV and sickle cell disease, which are known risk factors for anaemia, were excluded from the primary study.

Cigarette smoking was evaluated by self report as is usual in clinical practice, however underreporting of smoking is possible as cotinine levels—a sensitive marker of smoking tobacco—were not measured. Nevertheless, in pregnancy, self-reported tobacco use has been found to be a valid marker of tobacco exposure [38].

## Conclusion

In this low risk healthy pregnant population we found a low anaemia rate. The absence of a marital partner was a non-modifiable factor, albeit one which may reflect a variety of confounding factors, that should be considered for addition to the conceptual framework of anaemia’s determinants. Although not statistically significant, clinically, a trend towards a higher risk of adverse pregnancy outcomes was observed in women that were anaemic in early pregnancy.

## Supporting Information

S1 Table STROBE Statement—Checklist of items that should be included in reports of cohort studies. (DOC)
